# Pim1 kinase positively regulates myoblast behaviors and skeletal muscle regeneration

**DOI:** 10.1038/s41419-019-1993-3

**Published:** 2019-10-10

**Authors:** Yuantong Liu, Yue Shang, Zihan Yan, Hao Li, Zhen Wang, Zhen Liu, Zhenzhong Li

**Affiliations:** 1 Department of Anatomy, Shandong University School of Basic Medical Sciences, Jinan, 250012 China; 2 Department of Spine Surgery, Shenzhen People’s Hospital, Second Clinical Medical College of Jinan University, Shenzhen, 518020 China; 3 Department of Orthopaedics, Shandong University Qilu Hospital, Jinan, 250012 China

**Keywords:** Differentiation, Muscle stem cells, Regeneration

## Abstract

Adult skeletal muscle regeneration after injury depends on normal myoblast function. However, the intrinsic mechanisms for the control of myoblast behaviors are not well defined. Herein, we identified Pim1 kinase as a novel positive regulator of myoblast behaviors in vitro and muscle regeneration in vivo. Specifically, knockdown of Pim1 significantly restrains the proliferation and accelerates the apoptosis of myoblasts in vitro, indicating that Pim1 is critical for myoblast survival and amplification. Meanwhile, we found that Pim1 kinase is increased and translocated from cytoplasm into nucleus during myogenic differentiation. By using Pim1 kinase inhibitor, we proved that inhibition of Pim1 activity prevents myoblast differentiation and fusion, suggesting the necessity of Pim1 kinase activity for proper myogenesis. Mechanistic studies demonstrated that Pim1 kinase interacts with myogenic regulator MyoD and controls its transcriptional activity, inducing the expression of muscle-specific genes, which consequently promotes myogenic differentiation. Additionally, in skeletal muscle injury mouse model, deletion of *Pim1* hinders the regeneration of muscle fibers and the recovery of muscle strength. Taken together, our study provides a potential target for the manipulation of myoblast behaviors in vitro and the myoblast-based therapeutics of skeletal muscle injury.

## Introduction

Skeletal muscle has an ability of regeneration after injury due to its intrinsic stem cell reserve^1^. Skeletal muscle stem cells, also known as muscle satellite cells (SCs), reside between the plasma membrane and basal lamina in a quiescent state^2^. Once the skeletal muscle is damaged, the quiescent SCs are immediately activated to generate transient amplifying precursors called myoblasts^3^, which further differentiate and fuse either to form new multinucleated muscle fibers or to repair damaged parts of existing muscle fibers^1^. The regulation of these steps is closely associated with the sequential activation of a series of myogenic regulatory factors in SCs. Quiescent SCs highly express the transcriptional factor Pax7, while the activated SCs follow the specification to the myogenic lineage and begin to express Myf5, MyoD, myogenin, and MRF4, determining the entry of myoblast into the myogenic differentiation program, which eventually fuse with injured myofibers to accomplish regeneration^4^. Meanwhile, a part of proliferating myoblasts downregulates MyoD and maintains Pax7, returning the quiescent state and replenishing the stem cell pool^5^.

However, the intrinsic mechanisms that regulate these morphological and molecular events of muscle SCs remain poorly understood. So that in some special cases, such as extensive muscle trauma and severe myopathies, skeletal muscle will have difficulty initiating the regeneration program due to the exhaustion or functional defect of muscle SCs. Although some experimental studies and clinical trials based upon intramuscular myoblasts transplantation have shown encouraging results^6–9^, these outcomes are still of limited benefit ascribing to the poor ability of transplanted myoblasts to survival, expansion, and migrate^2,10–12^. Therefore, exploring the intrinsic mechanisms of SCs self-renewal, proliferation, differentiation, and fusion has vital application value for the manipulation of SCs behaviors in vitro, as well as the restart of muscle regeneration program in vivo.

Previous studies have shown that the functions of SCs are regulated by multiple signal pathways^13–15^. Among them, protein kinase, serving as the extremely important signal proteins in mammalian cells^16^, plays a crucial role in the regulation of SCs functions. For example, protein kinase ERK and JNK can facilitate SCs self-renewal^17,18^, and TAK can promote SCs proliferation and repress differentiation^19^, while p38 MAPK, Lkb1, and JAK can inhibit proliferation and accelerate differentiation of SCs^20–22^. It can be seen that the effect of these reported protein kinases on SCs is just reflected either in one state of SCs, or in boosting one state but restricting another state. Meanwhile, the proliferation, differentiation, and fusion of muscle SCs during regeneration are a staggered sequential process. Thus, the utilization of these reported protein kinase targets should be paid attention to the selection of the time window during muscle regeneration. Once the application opportunity is not well mastered, it will result in inadequate expansion or premature differentiation in myoblasts, which in turn limits the proper muscle repair. This problem virtually increases the difficulty and risk of clinical application of the reported kinase targets. Therefore, in this paper we will attempt to screen novel protein kinase that benefits multiple states of myoblasts, and reveal its effects on myoblast behaviors and muscle regeneration, providing potential target for the myoblast-based therapeutics of skeletal muscle injury.

## Materials and methods

### Bioinformatics

An expression profile microarray data of muscle injury model were downloaded from Gene Expression Omnibus (GEO) database. The wild-type control group and wild-type notexin (NTX) treatment group were selected and reanalyzed. The limma R-package of the R platform was used according to the user’s guide for screening differentially expressed genes (DEGs) between these two groups. The DEGs were identified with the following criterion: |fold change| ≥ 2 and *P*-value < 0.01. Then the DEGs were further annotated and the protein kinases among these DEGs were identified by gene ontology (GO) analysis using the Database for Annotation, Visualization, and Integrated Discovery (DAVID) v6.8 (https://david.ncifcrf.gov/). The volcano plot of the DEGs and the heat map of the differentially expressed protein kinases were drawn via the ggplots package in the R platform. The venn diagram was created through website Draw Venn Diagram (http://bioinformatics.psb.ugent.be/webtools/Venn/).

### Generation of Pim1 knockout mice

*Pim1* knockout (*Pim1*^−/−^) mice was obtained from Biomodel Organisms (Shanghai, China). Briefly, *Pim1*^−/−^ mice lacking exon I–VI of the *Pim1* gene were generated using CRISPR/Cas9-mediated genome editing in C57BL/6J embryonic stem cell. Heterozygous animals were bred to obtain homozygous *Pim1*^−/−^ mice. The *Pim1*^−/−^ mouse strain was genotyped by PCR using the primers 5′-CGGCGTTAGCGACCATTCTG-3′ and 5′-GGAAGAGGTGACAGGGACTTAA-3′. Mice were housed under specific pathogen-free conditions at 24 ± 2 °C with a 12:12 h light/dark cycle and ad libitum access to food and water. All animal experimental procedures were in accordance with the National Institutes of Health Guide for the Care and Use of Laboratory Animals and approved by the Ethical Committee for Animal Experimentation of the School of Medicine at Shandong University (Document No. LL-201602035).

### Muscle injury

The muscle injury mouse model was established by intramuscular injection of 10 μL of NTX (10 μg/ml, L8104, Latoxan) into the tibialis anterior (TA) muscle under pentobarbital sodium anesthesia (50 mg/kg, i.p.). The TA muscles were harvested and weighed at different time points post injection and then quick-frozen in liquid nitrogen and stored at −80 °C or immersed directly in 4% paraformaldehyde (PFA) until the time of molecular or histological analysis.

### Cell viability assay

C2C12 cell viability was determined using the CCK-8 kit in accordance with the manufacturer’s protocol. In brief, C2C12 cells were seeded on 96-well cell culture plates at a density of 5000 cells per well. At 24, 36, 48, and 60 h after seeding, 10 μL CCK-8 solution was added to 100 μL medium and incubated at 37 °C for 1 h. Then, the optical densities of the cells at 450 nm were measured with a Multiskan Mk3 microplate reader (Thermo Scientific). Cell viability was calculated as a proportion of the control group.

### Proliferation and apoptosis assays

To assay proliferation, C2C12 cell lines were incubated with EdU at a final concentration of 10 μM for 2 h before fixation and stained nuclei with Hoechst using the Click-iT EdU Alexa Fluor 555 Imaging kit (C10638, Invitrogen) according to the manufacturer’s protocol. To assay apoptosis, TUNEL staining was performed to monitor the cell apoptosis using the In Situ Cell Death Detection Kit (11684795910, Roche) in accordance with the manufacturer’s instructions. Images were then captured using an Olympus BX63 fluorescence microscope. Orange red EdU dots or green TUNEL dots located within the nucleus were defined as proliferative or apoptotic cells. The ratio of proliferative or apoptotic cells to total cells was calculated.

### Luciferase activity assay

For the MyoD transcriptional activity assay, C2C12 myoblasts in 24-well plate were transfected with expression plasmids for Pim1 K67M-Flag (200 ng), MyoD-HA (100 ng), the E-box-specific 4RE-luciferase reporter (4RE-luc) (300 ng), *MyoG* promoter (1444 bp)-luciferase reporter (*MyoG* pro-luc) (300 ng) and Renilla luciferase (RL) reporter (20 ng) using lipofectamine 2000 (Invitrogen). Transfected cells were induced to differentiate for 72 h. Then the cells were lysed and luciferase activity was measured for four times by a microplate luminometer (Centro LB 960, Berthold) with the Dual-Glo Luciferase Assay System (Promega). Firefly luciferase activity was normalized to the Renilla luciferase activity for each transfected well and the results were presented as a proportion of the activity of the basic luciferase vector (4RE-luc).

### Muscle force measurement

The function of TA muscles was evaluated by measuring in vivo muscle contraction in response to nerve stimulation. The mice were anaesthetized with pentobarbital sodium (50 mg/kg). The knee and paw were fixed in place to prevent movement from the contraction of other muscle groups and the distal tendon of the TA muscle was dissected and attached to a JZ101 tension transducer (Yuyan Instruments, Shanghai, China) using a silk ligature. Electrical stimulations were applied across two needle electrodes placed beneath the TA muscle to stimulate the tibial nerve. Capacity for force generation was evaluated by measuring the absolute maximal force that was generated during tetanic contractions in response to electrical stimulation (train of 55 Hz for 300 ms pulses). Data were collected with a Medlab Data Acquisition and Processing Systems (Yuyan Instruments). Force was normalized by muscle mass as an estimate of specific tetanic force.

### Statistical analysis

The statistical significance of differences between experimental groups was determined by a two-tailed Student’s *t*-test using SPSS 13.0 software^23^. The data are presented as the mean ± SD or mean ± SEM (*P < 0.05; **P < 0.01; and ***P < 0.001).

## Results

### Pim1 kinase is upregulated after skeletal muscle injury

**Fig. 1:**
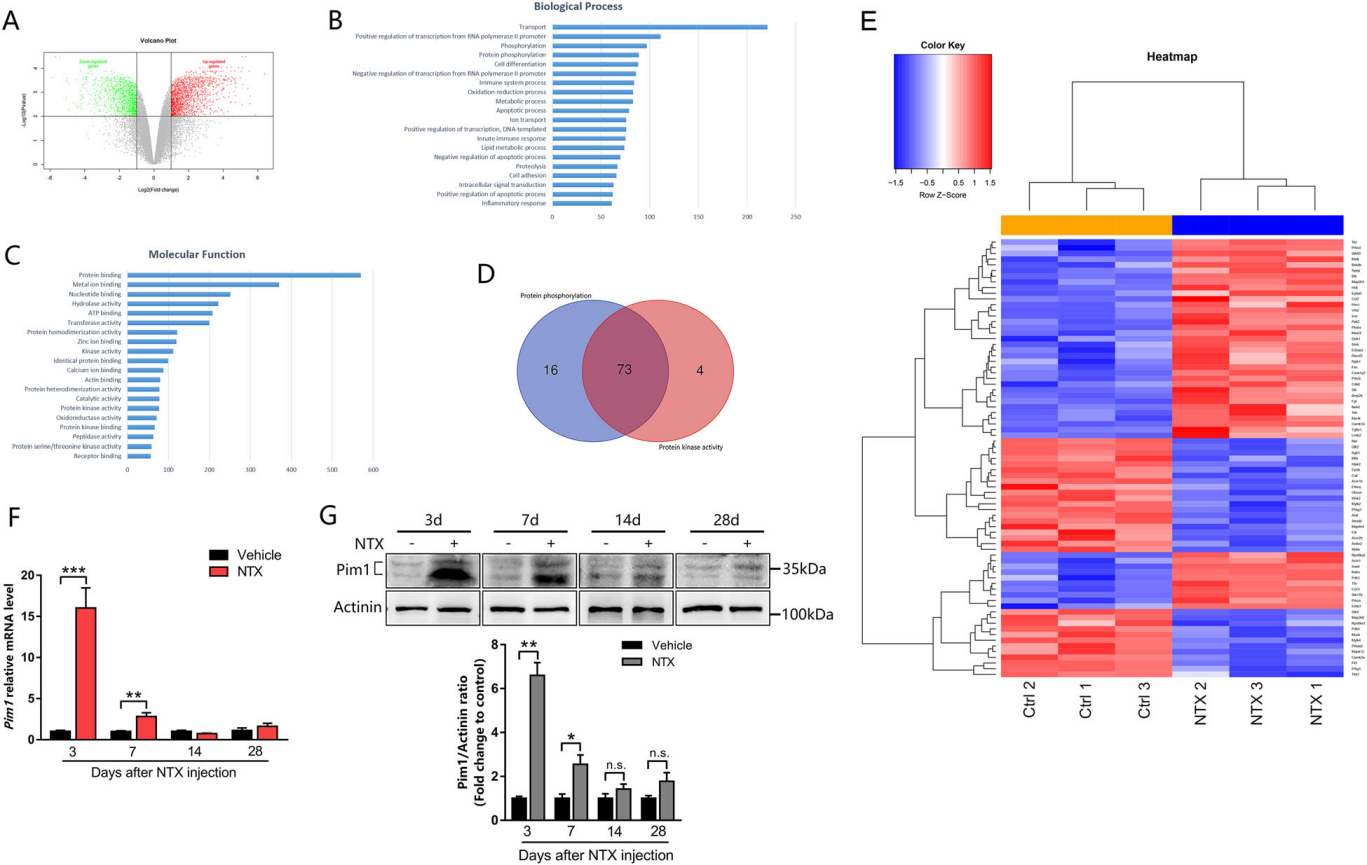
Pim1 kinase is upregulated after skeletal muscle injury. **a** Volcano plots of differentially expressed genes (DEGs) in normal muscle and damaged muscle samples in GSE67032. DEGs were selected by *P-*value < 0.01 and |log2 (fold change)| ≥ 1. The *x*‑axis corresponds to log2 (fold change), and *y*‑axis corresponds to −log10 (*P-*value). **b**, **c** Gene ontology (GO) analysis of identified DEGs. Top 20 biological process **b** and molecular function **c**. **d** Venn diagrams illustrating the number of common DEGs that participate in both protein phosphorylation and protein kinase activity. **e** Heat map of the 73 differentially expressed protein kinases that participate in protein phosphorylation in GSE67032. Red: upregulated kinases; blue: downregulated kinases. **f**, **g** qPCR and western blot analysis of Pim1 mRNA **f** and protein **g** levels in the TA muscle at 3, 7, 14, and 28 days after notexin (NTX) injection (*n* = 4). The data are shown as mean ± SEM. Independent-samples *t-*test. *P < 0.05; **P < 0.01; ***P < 0.001; n.s. = not significant

To screen novel protein kinases that regulate skeletal muscle regeneration, we firstly downloaded the expression profile microarray data GSE67032 from GEO database. The data of wild-type control group and wild-type NTX treatment group were reanalyzed for screening the DEGs by the limma R-package of the R platform. A total of 2703 genes were obtained, including 1634 upregulated and 1069 downregulated (Fig. 1a). According to GO enrichment analysis for biological process and molecular function, we got 89 genes that participate in protein phosphorylation and 77 protein kinases from the 2703 DEGs (Fig. 1b, c). Furthermore, 73 genes were obtained by taking the intersection of these two GO terms (Fig. 1d). Pim1 was one of the upregulated protein kinases modulating protein phosphorylation (Fig. 1e).

Next, we established a skeletal muscle injury mouse model by NTX intramuscular injection to verify the expression of Pim1. As previously described^24^, the 34-kDa isoform of Pim1 was visualized as two bands in whole-cell lysate immunoblots: the phosphorylated band of higher moiety and the unphosphorylated band of lower moiety. In our research, western blot and qPCR showed that both protein and mRNA expression of Pim1 were significantly upregulated in TA muscle at day 3 and day 7 after NTX injection, and returned to normal at day 14 and day 28 post-injection (Fig. 1f, g). Importantly, the time window of Pim1 upregulation in our study coincides with the peak of myoblast proliferation and differentiation^5^, suggesting that Pim1 kinase is likely involved in the process of myoblast mediated-skeletal muscle regeneration.

### Pim1 kinase is required for myoblast proliferation and survival in vitro

**Fig. 2:**
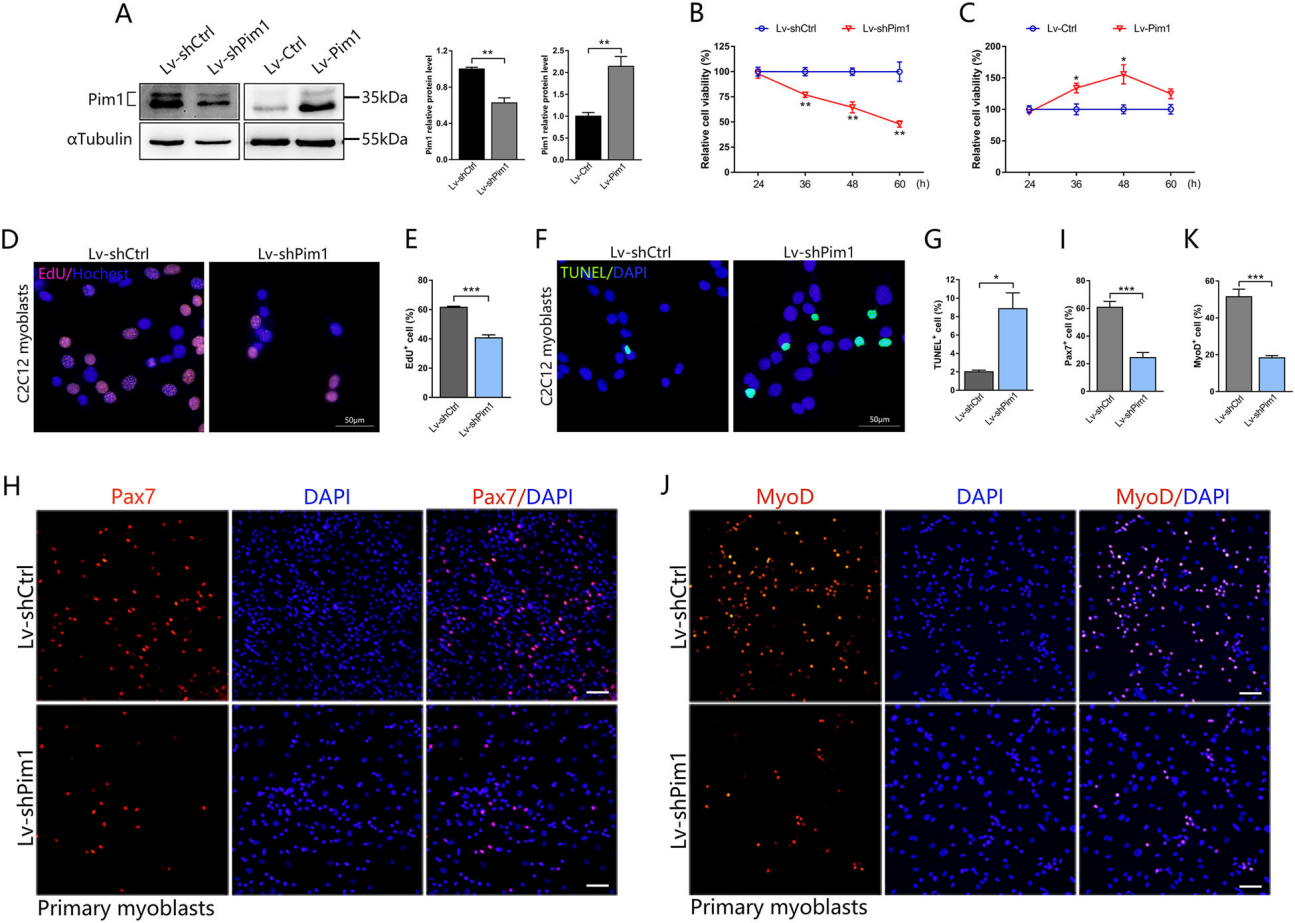
Pim1 kinase is required for myoblast proliferation and survival in vitro. **a** Expression and quantification of the Pim1 protein in C2C12 myoblasts after Lv-shPim1 or Lv-Pim1 infection (*n* = 3). αTubulin served as the internal reference. **b**, **c** CCK-8 assays were performed to determine the viability of C2C12 cells in which Pim1 had been silenced **b** or overexpressed **c**. The data were quantified as the percentage relative to the control (*n* = 3). **d**–**g** Representative immunofluorescence images of the C2C12 myoblasts after Lv-shPim1 infection labeled with EdU (orange red) **d** or TUNEL (green) **f**, and the percentage of EdU^+^ cells **e** (*n* = 5) or TUNEL^+^ cells **g** (*n* = 4). Hochest or DAPI (blue) labeled nuclei. Scale bar = 50 μm. **h**–**k** Representative immunofluorescence images of the primary myoblasts after Lv-shPim1 infection labeled with Pax7 (red) **h** or MyoD (red) **j**, and the percentage of Pax7^+^ cells **i** or MyoD^+^ cells **k** (*n* = 4). DAPI (blue) labeled nuclei. Scale bar = 50 μm. The data are shown as mean ± SEM. Independent-samples *t* test. *P < 0.05; **P < 0.01; ***P < 0.001

To gain a better understanding of the effect of Pim1 on myoblast behaviors, we established Pim1 low-expression and over-expression C2C12 myoblast cell lines by Lv-shPim1 or Lv-Pim1 infection. As expected, infection with Lv-shPim1 efficiently downregulated, while infection with Lv-Pim1 upregulated Pim1 protein level in C2C12 myoblasts (Fig. 2a). Lv-shPim1 infection inhibited the cell viability in a time-dependent manner, as shown by CCK-8 assay (Fig. 2b). Conversely, Pim1 overexpression enhanced the C2C12 myoblast viability (Fig. 2c). Meanwhile, the reduced cell viability after Lv-shPim1 infection was accompanied by a marked reduction of cell proliferation, as indicated by a decrease of the number of EdU^+^ cell (Fig. 2d, e). Meanwhile, we observed an increase in TUNEL^+^ cell (Fig. 2f, g) in Pim1 low-expression C2C12 myoblasts, suggesting that Pim1 knockdown in C2C12 myoblasts accelerated apoptosis. Altogether, these findings indicated that the proliferation and survival of C2C12 myoblasts are dependent on Pim1 kinase. Consistent with the results from C2C12 cell lines, Pim1 knockdown also restrained the proliferation of primary cultured myoblasts as demonstrated by a marked reduction of the proportion of Pax7^+^ and MyoD^+^ cell (Fig. 2h–k).

### Pim1 kinase is upregulated and translocated from cytoplasm into nucleus during myogenic differentiation

**Fig. 3:**
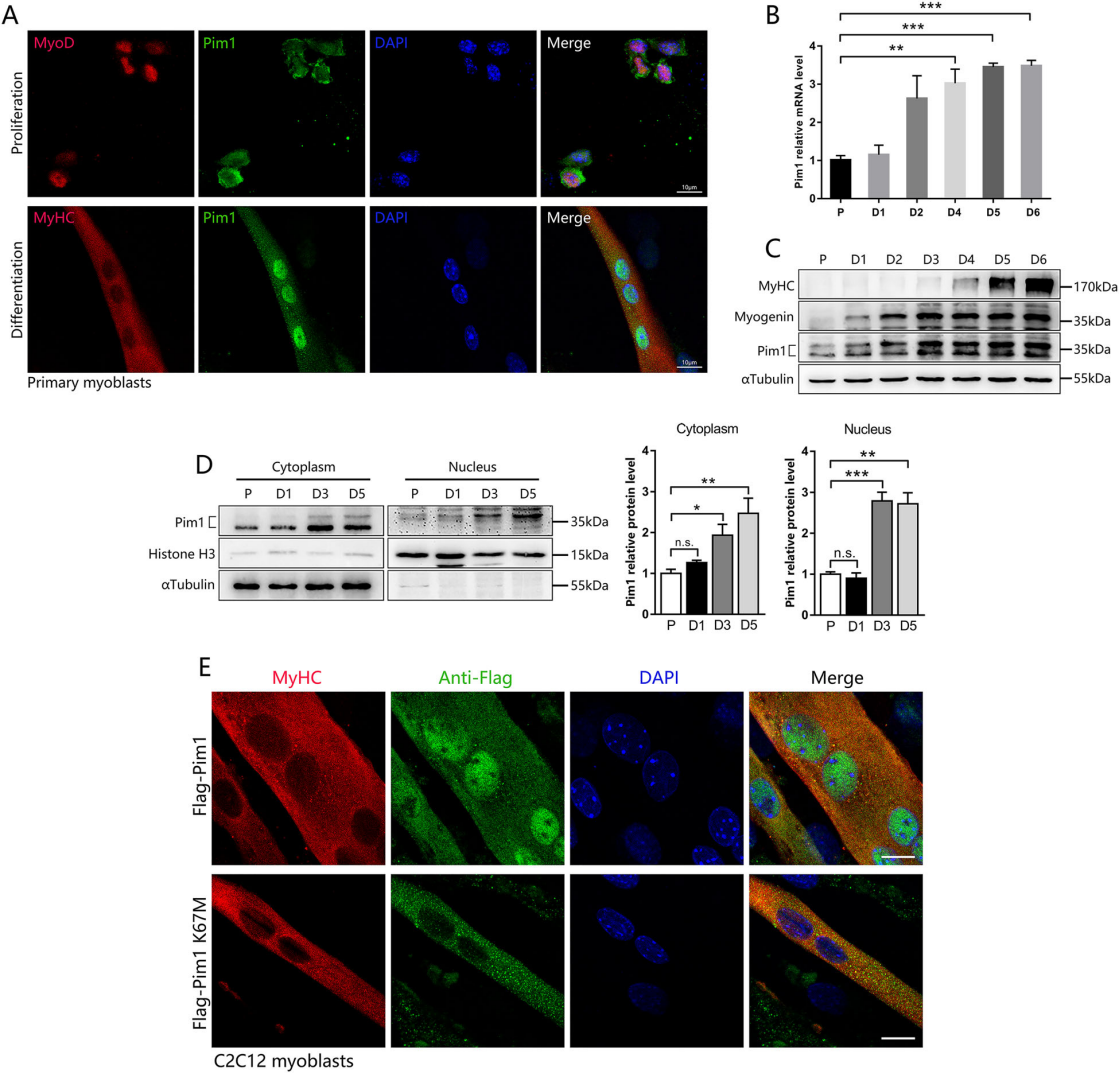
Pim1 kinase is upregulated and translocated from cytoplasm into nucleus during myogenic differentiation. **a** Immunofluorescence was performed to determine the subcellular localization of Pim1 kinase in proliferative and differentiated primary myoblasts. Green for Pim1 kinase; Red for MyoD or MyHC; Blue for nuclei labeled with DAPI. Scale bar = 10 μm. **b**, **c** qPCR and western blot analysis of Pim1 mRNA **b** and protein **c** levels during myogenic differentiation (*n* = 3). P proliferation, D differentiation. **d** Subcellular fractionation analysis showing the protein level of cytosolic Pim1 and nuclear Pim1 during myogenic differentiation (*n* = 4). P proliferation, D differentiation. **e** Confocal images for the analyses of subcellular localization of Pim1 in 5-day-differentiated C2C12 myotubes using anti-Flag (green) and anti-MyHC (red) antibodies. C2C12 myoblasts were transduced with the wild-type Pim1 plasmid and the kinase-inactive Pim1 K67M plasmid, and induced for differentiation. Scale bar = 10 μm. The data are shown as mean ± SEM. Independent-samples *t* test. *P < 0.05; **P < 0.01; ***P < 0.001; n.s. = not significant

To investigate the association of Pim1 kinase with myoblast differentiation, we first tested the localization of Pim1 kinase in proliferating and differentiated SCs by immunofluorescence. To our surprise, the Pim1 kinase was present in the cytoplasm during SCs proliferation, but was mostly transferred into the nucleus upon differentiation (Fig. 3a). Then, we analyzed the Pim1 expression in whole-cell lysate over the time course of differentiation. Notably, qPCR and western blot showed a gradual increase in Pim1 mRNA and protein during C2C12 cell differentiation (Fig. 3b, c). Subcellular fractionation study showed that the cytosolic Pim1 protein increased by 1.9-fold, while the nuclear Pim1 protein increased by 2.8-fold at day 3 post differentiation (Fig. 3d). Importantly, the nuclear Pim1 was visualized as the phosphorylated band of higher moiety in immunoblots while the cytosolic Pim1 was unphosphorylated (Fig. 3d). We further transduced the wild-type Pim1 plasmid and the kinase-inactive Pim1 K67M plasmid into C2C12 myoblasts. Immunofluorescence showed that the kinase-inactive Pim1 K67M was not transferred into the nucleus during differentiation (Fig. 3e), suggesting that the activity of Pim1 kinase is necessary for its nuclear translocation.

### Pim1 kinase in nuclei facilitates myoblast differentiation by mediating MyoD activity

**Fig. 4:**
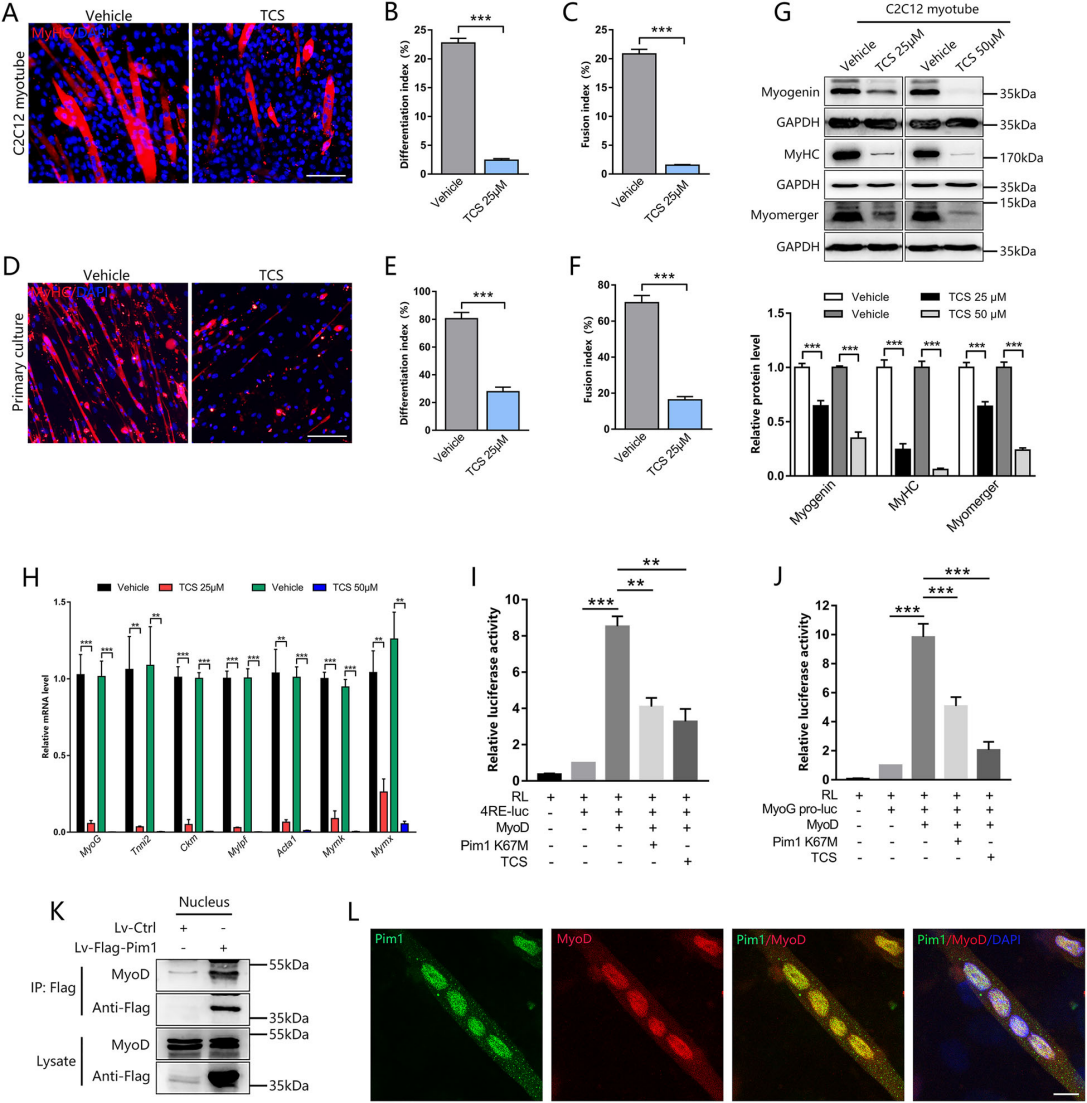
Pim1 kinase in nuclei facilitates myoblast differentiation by mediating MyoD activity. **a**–**c** Representative immunofluorescence images of the C2C12 myotubes after Pim1 inhibitor TCS (25 μM) treatment for 72 h **a**, and the quantification of differentiation index **b**, and fusion index **c** (*n* = 6). Red for MyHC; blue for nuclei labeled with DAPI. Scale bar = 100 μm. **d**–**f** Representative immunofluorescence images of the primary myotubes after TCS (25 μM) treatment for 72 h **d**, and the quantification of differentiation index **e** and fusion index **f** (*n* = 4). Red for MyHC; blue for nuclei labeled with DAPI. Scale bar = 100 μm. **g** Expression and quantification of the MyHC, myogenin, and myomerger protein in C2C12 myotubes after TCS (25, 50 μM) treatment for 72 h (*n* = 8). GAPDH served as the internal reference. **h** qPCR analysis of the muscle-specific genes, such as *MyoG*, *Tnni2*, *Ckm, Mylpf*, *Acta1*, *Mymk*, and *Mymx* in C2C12 myotubes after TCS (25, 50 μM) treatment for 72 h (*n* = 4–5). **i**, **j** Luciferase assay was performed to determine the effect of Pim1 kinase on the activity of the 4RE (MyoD-binding core sequence) **i** (*n* = 5) and *MyoG* promoter **j** (*n* = 8) in C2C12 cells transfected with indicated vector. The data were presented as a proportion of the activity of the basic luciferase vector (4RE-luc). **k** Co-immunoprecipitation (Co-IP) using anti-Flag antibody revealing the interaction between Pim1 and MyoD in C2C12 myotubes transfected with Lv-Flag-Pim1. **l** Confocal images showing the colocalization between Pim1 (green) and MyoD (red) in C2C12 nuclei (blue, DAPI-labeled) on 4 days post-differentiation. Scale bar = 10 μm. The data are shown as mean ± SEM. Independent-samples *t*-test for **b**, **c**, **e**, **f**, **g**, **h**; Paired-samples *t-*test for **i**, **j**. **P < 0.01; ***P < 0.001

To further evaluate the requirement of Pim1 kinase activity in nuclei on myoblast differentiation in vitro, we treated C2C12 myoblasts and primary myoblasts with Pim1 kinase inhibitor TCS during the induction of differentiation. Immunofluorescence showed that inhibition of Pim1 activity did impair myogenic differentiation and fusion of C2C12 cells and primary myoblasts, as indicated by a lower differentiation index and fusion index compared to control (Fig. 4a–f). Meanwhile, the expression of myogenic regulatory factor myogenin, muscle structural protein MyHC, and fusion-related protein myomerger was restrained upon TCS treatment in a dose-dependent manner (Fig. 4g). Similarly, qPCR results showed a significant decrease in the mRNA level of muscle-specific genes (*MyoG, Tnni2, Ckm, Mylpf, Acta1, Mymk*, and *Mymx*) involved in myogenic differentiation and fusion after TCS treatment (Fig. 4h).

To investigate the mechanisms by which Pim1 kinase promotes myogenic differentiation, we focused on MyoD, the basic helix–loop–helix (bHLH) myogenic regulatory factor that drives the specification and differentiation of myoblasts into myotubes^25,26^. Given that the inhibition of Pim1 activity did not reduce the protein expression of MyoD in the differentiated C2C12 cells (Fig. S1), while the mRNA expression of MyoD-targeting genes (*MyoG*, *Ckm*, *Tnni2*) was significantly inhibited (Fig. 4h). Therefore, we hypothesized that Pim1 kinase activity contributes to the MyoD-dependent transcriptional activation. To test this hypothesis, we analyzed the activity of 4RE-luc (firefly luciferase reporter plasmid controlled by MyoD-binding core sequence) and *MyoG* pro-luc (firefly luciferase reporter plasmid controlled by *MyoG* promoter) by luciferase reporter assay in C2C12 cells. As shown in Fig. 4i, j, both kinase-inactive Pim1 K67M plasmid and Pim1 inhibitor TCS significantly inhibited the activity of 4RE-luc and *MyoG* pro-luc. To explore how Pim1 affects the transcriptional activity of MyoD, we performed co-immunoprecipitation (Co-IP) in C2C12 cells transfected with Lv-Flag-Pim1. As shown in Fig. 4k, Co-IP experiment of nuclear protein using Flag antibody revealed the interaction between Pim1 and MyoD on 4 days post-differentiation. Furthermore, confocal images showed a strong colocalization between Pim1 and MyoD in the nucleus of C2C12 and primary myotube (Fig. 4l, S2). Taken together, these data suggested that Pim1 kinase combines with myogenic regulator MyoD in myonuclei and facilitates its transcriptional activity, inducing the expression of muscle-specific genes, which consequently promotes myogenic differentiation.

### Pim1^−/−^ mice exhibit no myopathy but a deficit of muscle regeneration

**Fig. 5:**
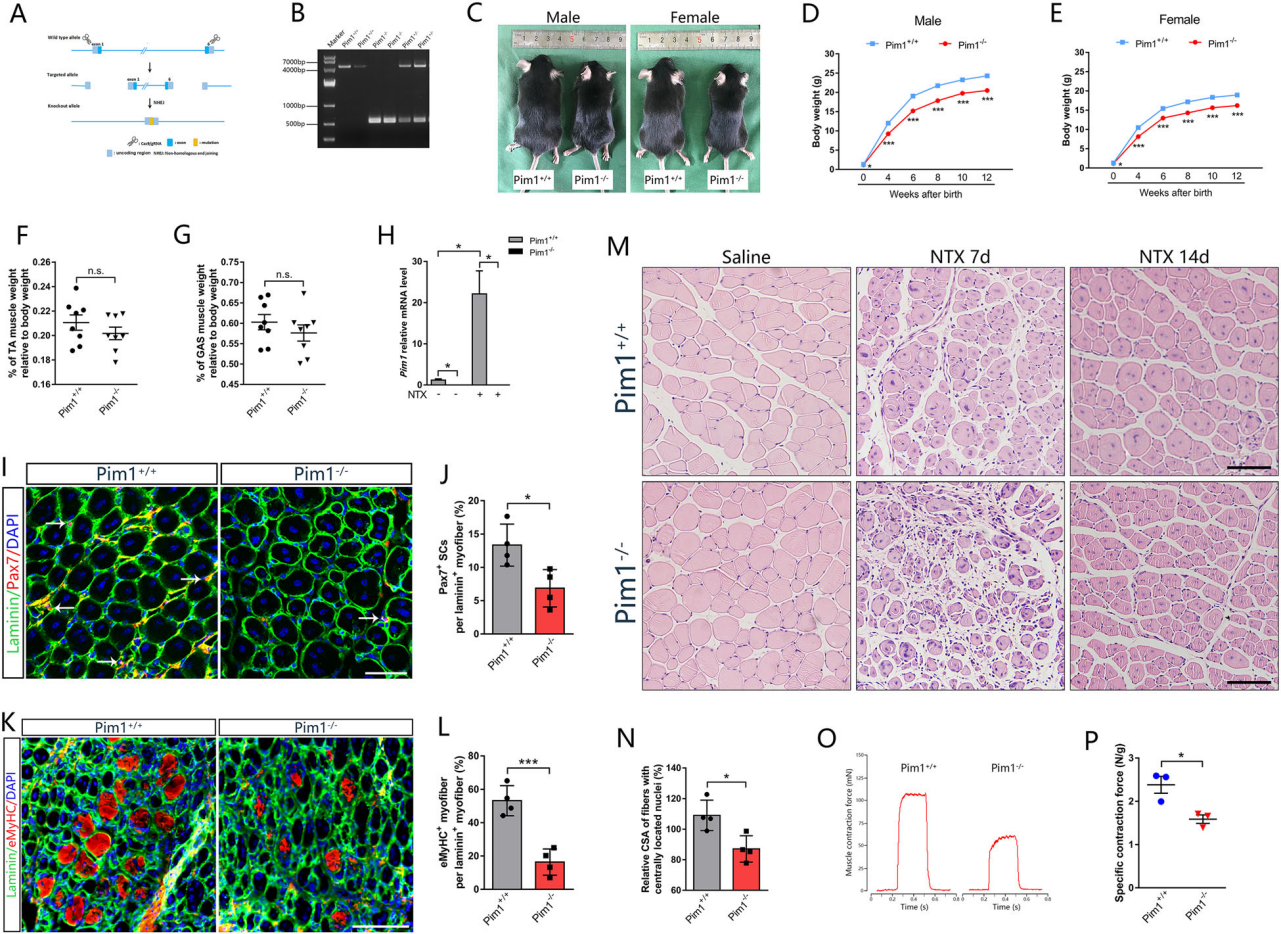
Deletion of *Pim1* exhibit no myopathy but a deficit of muscle regeneration. **a** Schematic showing the generation of *Pim1*^−/−^ mice through CRISPR/Cas9-mediated genome editing in C57BL/6J background. **b** Genotyping of *Pim1*^−/−^ mouse strain by PCR. Wild type: a 4684 bp fragment; Heterozygous: a 4684 bp fragment and a 536 bp fragment; Homozygous: a 536 bp fragment. **c** Representative image of *Pim1*^+/+^ and *Pim1*^−/−^ mice at 12-week old. **d**, **e** Growth curve of *Pim1*^+/+^ and *Pim1*^−/−^ mice from birth to 12-week old (*n* = 10−45 mice for each group). Male **d**; Female **e**. **f**, **g** Percentage of TA **f** and GAS **g** muscle weight relative to body weight in *Pim1*^+/+^ and *Pim1*^−/−^ mice (*n* = 8). **h** qPCR analysis of *Pim1* mRNA level in TA muscle after 3 days NTX-injection in *Pim1*^+/+^ and *Pim1*^−/−^ mice (*n* = 6). **i**, **j** Representative immunofluorescence images **i** of the TA muscle transverse section in *Pim1*^+/+^ and *Pim1*^−/−^ mice labeled with Pax7 (red) and laminin (green), and the percentage **j** of Pax7^+^ cells relative to laminin^+^ fibers (*n* = 4). DAPI (blue)-labeled nuclei. Scale bar = 50 μm. **k**, **l** Representative immunofluorescence images **k** of the TA muscle transverse section in *Pim1*^+/+^ and *Pim1*^−/−^ mice labeled with embryonic MyHC (eMyHC) (red) and laminin (green), and the percentage **l** of eMyHC^+^ fibers relative to laminin^+^ fibers (*n* = 4). DAPI (blue) labeled nuclei. Scale bar = 100 μm. **m** H&E staining of the TA muscle transverse section in *Pim1*^+/+^ and *Pim1*^−/−^ mice at 7 and 14 days after NTX injection. Scale bar = 100 μm. **n** Percentage of the cross-sectional area (CSA) of fibers with centrally located nuclei relative to contralateral in *Pim1*^+/+^ and *Pim1*^−/−^ mice at 14 days after NTX injection (*n* = 4). **o**, **p** Muscle contractile force was measured at 14 days after NTX injection in *Pim1*^+/+^ and *Pim1*^−/−^ mice. **o** Representative contractile force. **p** Quantification of muscle contractile forces (*n* = 3). The data are shown as mean ± SEM for **d**, **e**, **f**, **g**, **h**, **p** and mean ± SD for **j**, **l**, **n**. Independent-samples *t* test. *P < 0.05; ***P < 0.001; n.s. = not significant

We generated *Pim1* knockout (*Pim1*^−/−^) mice through the ablation of exon I–VI of *Pim1* gene by using CRISPR/Cas9-mediated gene editing system (Fig. 5a). All mice were viable and were identified by tail genotyping (Fig. 5b). Firstly, we analyzed the body weight, muscle weight, and morphology of *Pim1*^−/−^ mice and wild type (*Pim1*^+/+^) mice in age-matched groups. In a 12-week follow-up, the body weight of *Pim1*^−/−^ mice was significantly lower than that of control littermates (Fig. 5c–e), showing an overall growth retardation. But the proportion of the wet weight of TA and GAS muscles relative to body weight was not different from that of control littermates (Fig. 5f, g). H&E staining also showed that *Pim1*^−/−^ mice has no severe myopathy characterized by central nucleated myofibers from birth to 12-week old (Fig. S3).

To determine whether deletion of *Pim1* affects skeletal muscle regeneration in vivo, we established a NTX-induced muscle injury model with 10–12-week-old *Pim1*^+/+^ and *Pim1*^−/−^ male mice. Strikingly, a virtually complete absence of the mRNA and protein of Pim1 was observed in isolated TA muscles from both saline and NTX treated *Pim1*^−/−^ mice (Fig. 5h, S4), which ensured that *Pim1* gene was deleted in TA muscle. Firstly, the SCs in *Pim1*^−/−^ mice showed a striking block of cell expansion 7 days after injury, as reflected by a marked reduction of the number of Pax7^+^ cell (*Pim1*^+/+^ 13.34 ± 3.15%, *Pim1*^−/−^ 6.86 ± 2.81%) (Fig. 5i, j). The frequency of embryonic MyHC (eMyHC, an early marker of muscle regeneration) positive myofibers within laminin staining was dramatically decreased in TA muscle of Pim1^−/−^ mice at 5 days post injury (Fig. 5k, l). Next, we performed histological methods to analyze the effect of Pim1 on muscle fiber morphology during regeneration. Compared with *Pim1*^+/+^ littermates, *Pim1*^−/−^ mice showed an impaired muscle regeneration, as indicated by a smaller CSA of fibers with centrally located nuclei at 14 days post injury (Fig. 5m, n). These changes reflect the remodeling of muscle architecture that accompanies the retarded regeneration in *Pim1*^−/−^ mice, as the relative weight of injured TA muscle in *Pim1*^−/−^ mice did not decrease during this time period (Fig. S5). In addition to morphology, we then investigated whether the impaired myofiber regeneration in Pim1-deficient mice altered the function of muscle contraction. We observed a marked reduction in muscle contraction as demonstrated by a striking decrease in specific tetanic force in the damaged TA muscle of Pim1^−/−^ mice at day 14 after injury (Fig. 5o, p). Altogether, these findings showed that deletion of *Pim1* in skeletal muscle restricts myofiber regeneration morphologically and retards strength recovery functionally upon muscle injury, emphasizing the requirement of Pim1 for proper skeletal muscle repair.

## Discussion

Pim1, a serine/threonine protein kinase, is a member of the proviral integration site for moloney murine leukemia virus (Pim) kinase family composed of three different isoforms (Pim1, Pim2, Pim3)^27^. According to molecular weight and cellular localization, Pim1 kinase contains two isoforms, a long 44 kDa isoform, located in cell membrane and a short 34 kDa isoform, localized in cytoplasm and nucleus^28,29^. Pim1 kinase is constitutively active when expressed in cells, and its activity is directly correlated with its expression level. Additionally, the posttranslational modification such as phosphorylation or dephosphorylation also affects Pim1 activity^29,30^.

Functionally, studies have well proven that the 34 kDa Pim1 plays a crucial role in a wide range of cell processes and diseases, such as cell proliferation, apoptosis, mitochondrial integrity, cellular senescence, myocardial injury, lupus nephritis and Alzheimer's disease through interaction, stabilization, and phosphorylation of many downstream targets^29,31–38^. Importantly, as a direct target gene of JAK/STAT-signaling pathway^21,39,40^, Pim1 kinase is also involved in immune regulation and inflammatory response. Pro-inflammatory factors enhance Pim1 expression, and inhibition of Pim1 activity reduces the production of inflammatory factors and chemokines^41^. Furthermore, inflammation engages in almost all biological processes in mammals. So Pim1 kinase exerts its effects likely by mediating inflammatory response. Meanwhile, emerging evidences demonstrate that muscle regeneration after injury entail appropriate immune responses and inflammatory signals that orchestrate efficacious muscle SCs functions^7,19,21,42–45^. Consequently, based on the above reasoning, we hypothesized that Pim1 kinase may be an important modulator for SCs-mediated skeletal muscle regeneration.

In this study, we found that Pim1 kinase is upregulated in the process of muscle regeneration through the expression profile DEGs screening between normal muscle and injured muscle, bioinformatics analysis, and experimental verification of muscle injury mouse model. This upregulation occurs on day 3 and 7 after muscle injury. Interestingly, evidence has demonstrated that the production of many inflammatory factors and chemokines in skeletal muscle is also increased sharply within 3 days after injury^7,42,44^. So we speculated that Pim1 upregulation after muscle injury may be caused by the stimulation of inflammatory factors and the activation of JAK/STAT-signaling pathway, but this inference needs further experimental evidence. More importantly, the peak of SCs proliferation is also at 3–4 days post-injury, then SCs enter the stage of differentiation and fusion at 6–14 days post-injury^5^. It can be seen that the time window of Pim1 upregulation coincides with the peak period of SCs proliferation and differentiation, suggesting that Pim1 kinase is likely involved in SCs myogenic lineage progression.

**Fig. 6:**
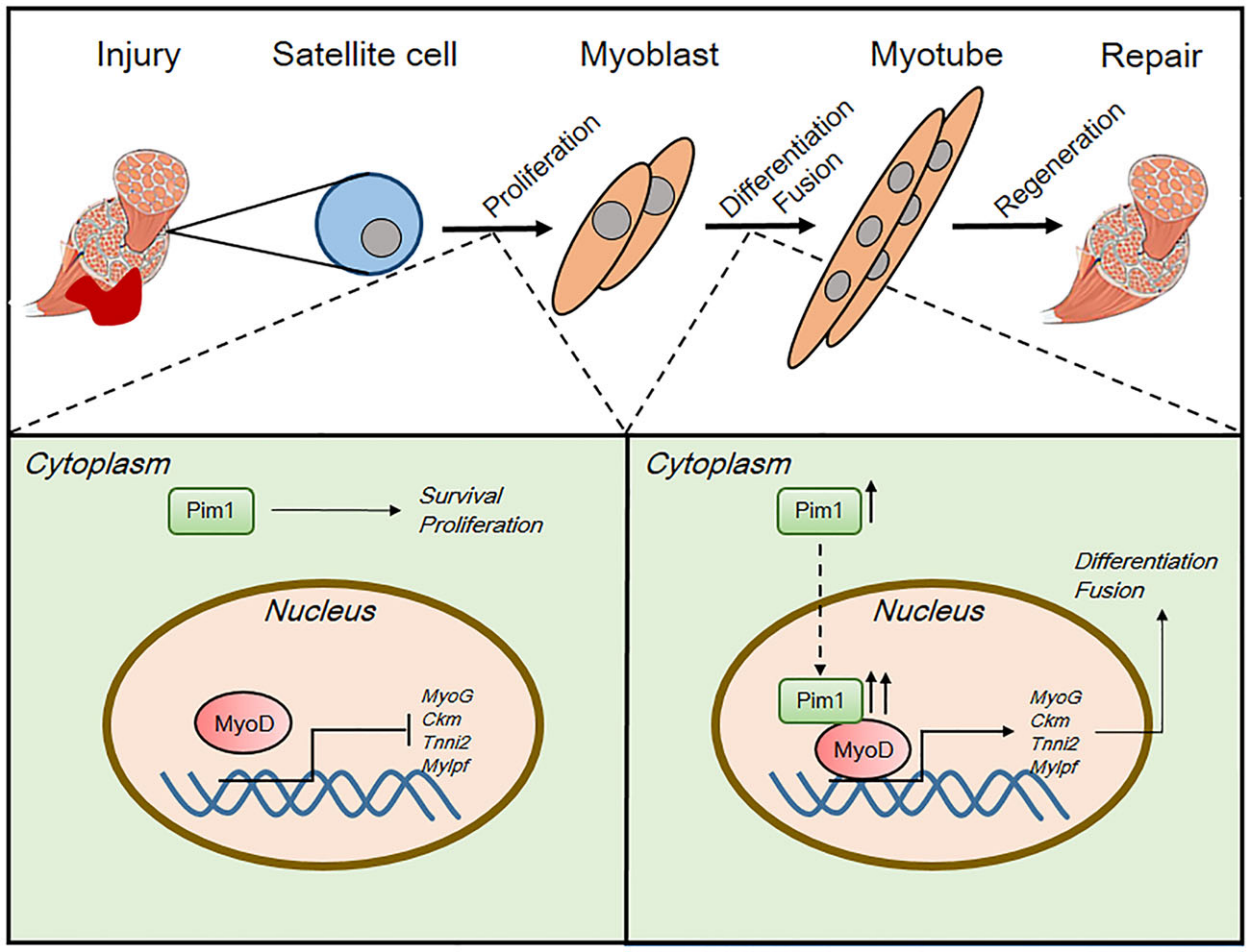
Schematic showing the regulation patterns of Pim1 kinase on myoblast and MyoD transcriptional activity during muscle regeneration. First, Pim1 kinase is required for myoblast survival and proliferation. Second, Pim1 kinase is upregulated and translocated from cytoplasm into nucleus during myogenic differentiation. Third, Pim1 kinase interacts with myogenic regulator MyoD, controls the transcriptional activity of MyoD, inducing the expression of muscle-specific genes, which consequently promotes the myogenic differentiation and fusion

Next, we established muscle injury model on *Pim1*^−/−^ mice to investigate the role of Pim1 kinase in skeletal muscle regeneration. It was found that deletion of *Pim1* shows a significant decrease in the CSA of muscle fibers with centrally located nuclei, implying that Pim1 kinase is vital for normal muscle regeneration. Since SCs are the ultimate executors of myofiber regeneration, we further explored the effects of Pim1 kinase on SCs behaviors at various stages. Consistent with the key role of Pim1 kinase in promoting proliferation and inhibiting apoptosis, it was found in this paper that Pim1 knockdown significantly restrained the proliferation and accelerated the apoptosis of myoblasts in vitro, indicating the necessity of Pim1 for myoblast survival and amplification (Fig. 6). In addition, we found that the expression level and subcellular localization of Pim1 changes over the course of myogenesis. Pim1 predominantly localizes to the cytoplasm of proliferating myoblast, but it is upregulated and translocated to the nucleus upon differentiation (Fig. 6). Moreover, in differentiated myotube, the nuclear Pim1 is phosphorylated while the cytosolic Pim1 is unphosphorylated. Coincidentally, a recent study has highlighted that the functional effect of Pim1 depends upon intracellular localization in human cardiac progenitor cells^46^. Thus, these evidences suggested that the upregulation of phosphorylated Pim1 in myocyte nucleus is likely associated with myogenic differentiation. By using Pim1 kinase inhibitor, we proved that inhibition of Pim1 activity prevents myoblast differentiation and fusion, suggesting that Pim1 kinase activity is required for the proper myogenesis (Fig. 6).

With respect to the underlying mechanism, previous study has confirmed that Pim1 contributes to bHLH transcription factor c-Myc-dependent transcriptional activation^47^. Interestingly, myogenic regulator MyoD is also a transcription factor with bHLH domain^25,26^. And, the direct targets of MyoD are downregulated upon Pim1 inhibition in our study. Therefore, we speculated that Pim1 kinase facilitates myoblast differentiation likely by mediating the transcriptional activity of MyoD. By Co-IP experiment and luciferase activity assay, we showed that Pim1 interacts with the key myogenic regulator MyoD, and either the transfection of kinase-inactive Pim1 plasmid, or the application of Pim1 inhibitor significantly inhibited the MyoD-dependent transcriptional activation, suggesting that Pim1 kinase activity facilitates myoblast differentiation to some extent by controlling the transcriptional activity of the bHLH myogenic regulator MyoD.

In summary, we demonstrated for the first time that Pim1 kinase is a positive modulator of myoblast functions and skeletal muscle regeneration, which will provide new ideas and experimental basis for the myoblast-based therapeutic strategies of skeletal muscle injury.

## Supplementary information

Supplementary materials and methods

Fig S1

Fig S2

Fig S3

Fig S4

Fig S5

Table S1

Supplemental Fig Legends - clean
