# Supplementary material for: Pim1 kinase positively regulates myoblast behaviors and skeletal muscle regeneration
Source: Cell Death Dis. 2019 Oct 10;10(10):773. doi: 10.1038/s41419-019-1993-3 (PMC6787030; doi:10.1038/s41419-019-1993-3)
Supplement: Supplementary file 1 — Supplementary materials and methods [file 41419_2019_1993_MOESM1_ESM.doc]

**Supplementary materials and methods**

**C2C12 cell culture and treatment**

C2C12 myoblasts were maintained in growth medium (GM) consisting of 20% foetal bovine serum (FBS) and 1% penicillin-streptomycin (Gibco) in DMEM-high glucose. For the induction of differentiation and fusion, the medium was replaced with differentiation medium (DM) supplemented with 2% horse serum (Gibco) and 1% penicillin-streptomycin in DMEM-high glucose until the cells reached 95% confluence. For Pim1 kinase inhibitor treatment, two-day-differentiated C2C12 cells were treated with 25μM and 50μM TCS PIM-1 I (TCS, MedChemExpress) or DMSO control for 72h.

**Primary myoblasts culture**

Hind limb muscles of 6-8w old male mice were isolated and excess connective tissues and fat were removed in sterile D-Hanks buffer. Muscles were minced into coarse slurry and digested for 1h at 37°C using 0.1U/ml collagenase and 0.8U/ml dispase (Roche). The digested slurry was passed through a 70-μm mesh filter to remove undigested tissue. Then, the filtrate was centrifuged at 1000rpm for 5min, and the cell pellet was resuspended in myoblast medium (Ham’s F-10 medium with 20% FBS supplemented with 10ng/ml of bFGF) and pre-plated in a 10cm plastic dish for 2h to allow fibroblasts to attach. After 2h, the cell suspension (enriched in slowly attaching satellite cells) was transferred to a new collagen-coated plate. Primary myoblasts were maintained in a 1:1 ratio of myoblast medium and GM.

**Lentivirus infection**

Mouse Pim1 shRNA lentivirus (Lv-shPim1) and Pim1 overexpression lentivirus (Lv-Pim1) (Genechem, Shanghai, China) were added to C2C12 cells or primary myoblasts at 30-40% confluency according to manufacturer’s instructions. The targeting sequence of the shPim1 was as follows: 5’-GTCATTAGACTTCTGGACT-3’. Cultures were selected by treating puromycin (5μg/ml) to create stable cell lines beginning 48h after transduction.

**Quantitative PCR (qPCR)**

Total RNA was isolated from frozen muscles or C2C12 cells using TRIzol (Invitrogen) according to the manufacturer’s protocol, and equal amounts of total RNA (1μg) were reverse-transcribed. The abundance of mRNA was detected by a realplex Mastercycler (Eppendorf) with SYBR Premix Ex Taq (Takara). The quantity of mRNA was analysed using the 2^-ΔΔCt^ method. All data were normalized to GAPDH, which was purchased commercially (Sangon Biotech). The mouse primers used in this study are listed in Table S1.

**Protein extraction and western blot**

Total protein extraction and western blot was performed as described previously^23^. For nuclear and cytosolic extraction, the C2C12 cells were harvested and subcellular fractionation was performed with a NE-PER nuclear and cytoplasmic extraction kit (78835, Thermo Scientific) according to the manufacturer’s protocol. The following commercially available primary antibodies were used: Rabbit anti-Pim1 (1:500, ab75776), rabbit anti-GAPDH (1:5000, ab181602), rabbit anti-Histone H3 (1:1000, ab1791), rabbit anti-actin (1:1000, ab46805), mouse anti-αTubulin (1:1000, ab7291), and mouse anti-Actinin (1:1000, ab9465) antibodies were from Abcam (Cambridge, UK). Mouse anti-myogenin (1:200, F5D), mouse anti-MyHC (1:1000, MF20) antibodies were from the Developmental Studies Hybridoma Bank (DSHB, Iowa, IA). Mouse anti-Pim1 (1:200, sc-374116), mouse anti-MyoD (1:200, sc-32758) antibodies were from Santa Cruz (Dallas, TX). Sheep anti-ESGP (Myomerger, 1:100, AF4580) antibody was from R&D Systems (Minneapolis, MN). Mouse anti-Flag (1:1000, F1804) antibody were from Sigma (St. Louis, MO).

**Co-immunoprecipitation (Co-IP)**

C2C12 cells were transfected with Lv-Flag-Pim1 or Lv-Ctrl. Then, nuclear extracts of four-day-differentiated C2C12 myotubes were pretreated with a mouse IgG and protein A/G mixed magnetic beads (Millipore) for 4h and then incubated with mouse anti-Flag (F1804, Sigma) antibody overnight with rotation, followed by the addition of 30µl of magnetic beads to the protein extracts for another 4h at 4°C. The beads were washed five times with PBST and resuspended in 30μl of 2× Sample Buffer (Sigma). Then, the samples were denatured at 98°C for 5min and analysed by western blot.

**Histology**

Muscles immersed in 4% PFA were placed in the Optimal Cutting Temperature cryoprotectant (Sakura) or embedded in paraffin. Subsequently, the samples were cryosectioned at a 10-μm thickness for further immunofluorescence analysis. The samples embedded in paraffin were sectioned into transverse sections with a thickness of 6μm, followed by haematoxylin and eosin (H&E) staining and examination under an Olympus BX63 microscope (Tokyo). H&E images were measured using ImageJ software for muscle fiber cross-sectional area (CSA) analysis.

**Immunofluorescence**

The cryosections of TA muscle or myoblasts were fixed in 4% PFA for 40 min and permeabilized with 0.3% Triton X-100 in PBS for 30min at room temperature. Blocking was performed with 10% goat serum in PBS for 2h. For staining, the samples were incubated with the following primary antibodies: Rabbit anti-Pim1 (1:100), mouse anti-MyoD (1:100), mouse anti-Pax7 (1:10), mouse anti-MyHC (1:200), mouse anti-embryonic MyHC (1:200), rabbit anti-laminin (1:500, L9393, Sigma), rabbit anti-Flag (1:500, F7425, Sigma) for overnight at 4°C, followed by incubation with fluorescence-labelled secondary antibodies (1:200 dilution) for 2h at 37°C. Fluorescence images were collected on an Olympus BX63 fluorescence microscope or a Zeiss LSM 780 laser scanning confocal microscope.
