# Supplementary figures and images for: Pim1 kinase positively regulates myoblast behaviors and skeletal muscle regeneration
Source: Cell Death Dis. 2019 Oct 10;10(10):773. doi: 10.1038/s41419-019-1993-3 (PMC6787030; doi:10.1038/s41419-019-1993-3)

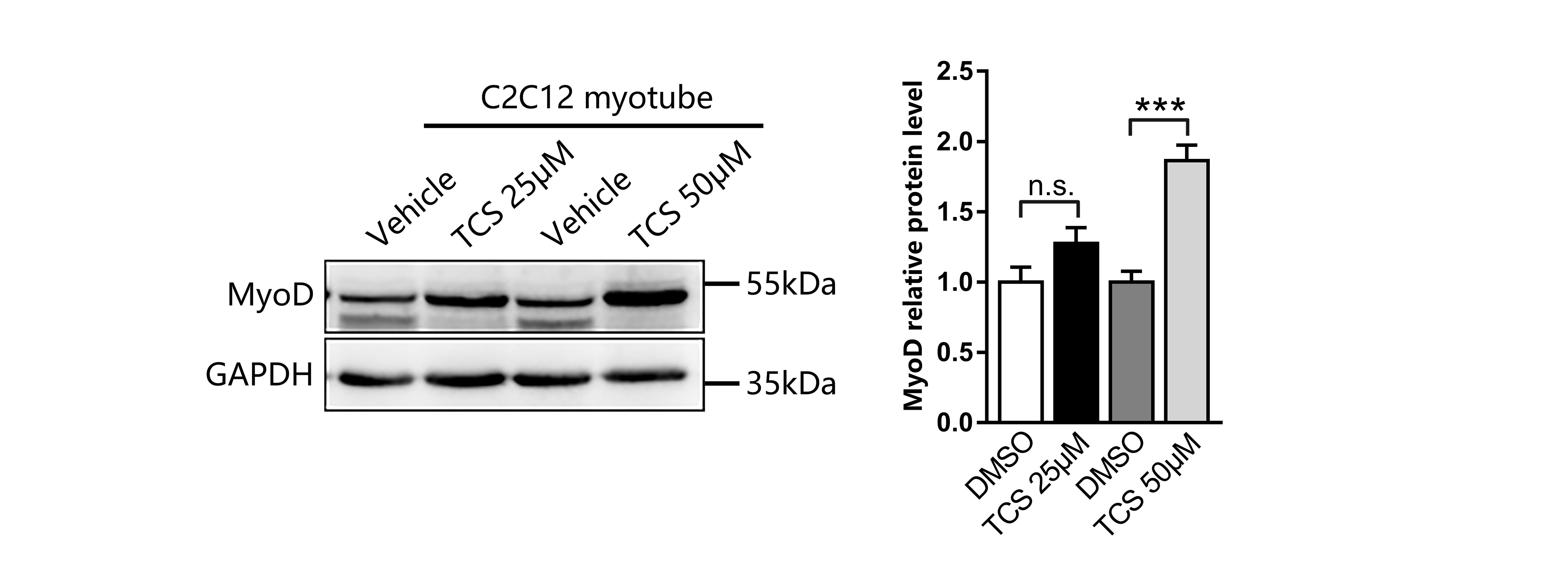

Supplement: Supplementary file 2 — Fig S1 [file 41419_2019_1993_MOESM2_ESM.tif]

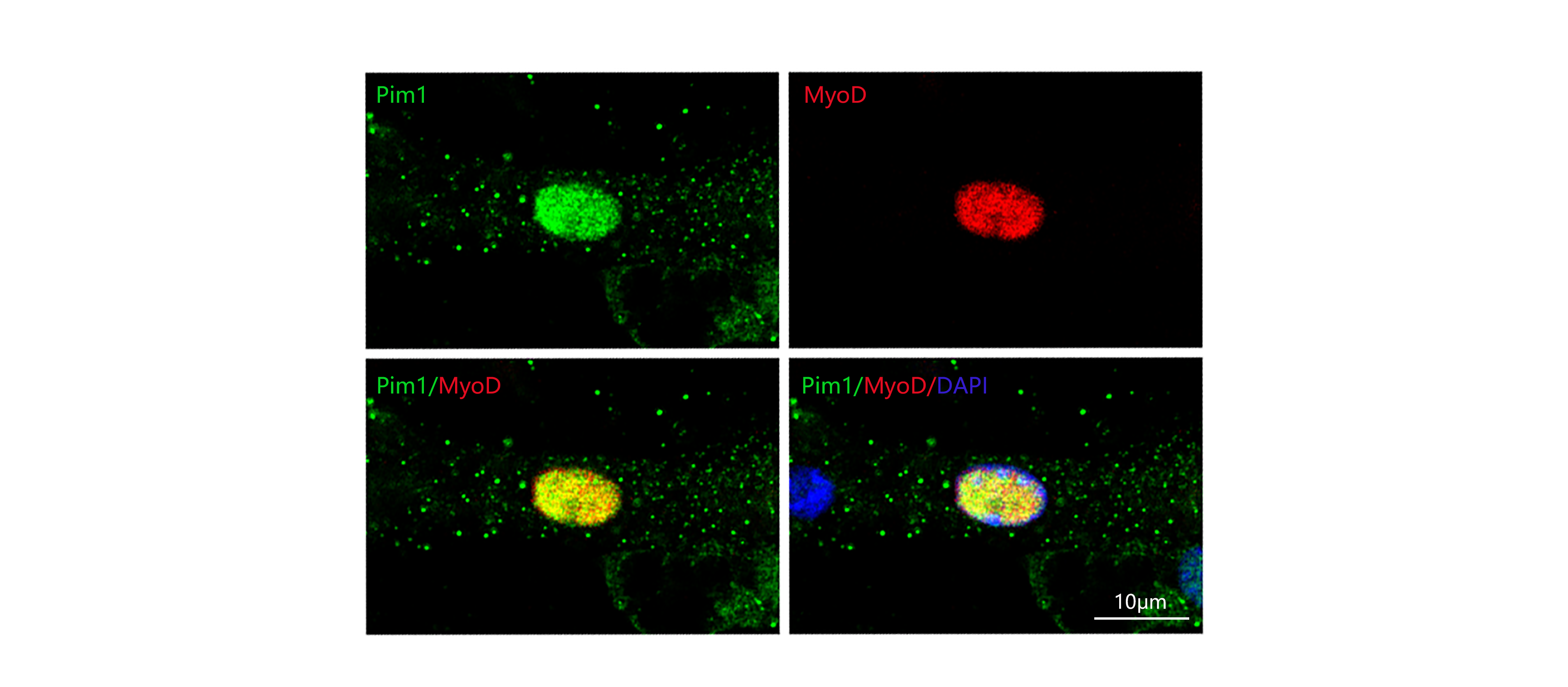

Supplement: Supplementary file 3 — Fig S2 [file 41419_2019_1993_MOESM3_ESM.tif]

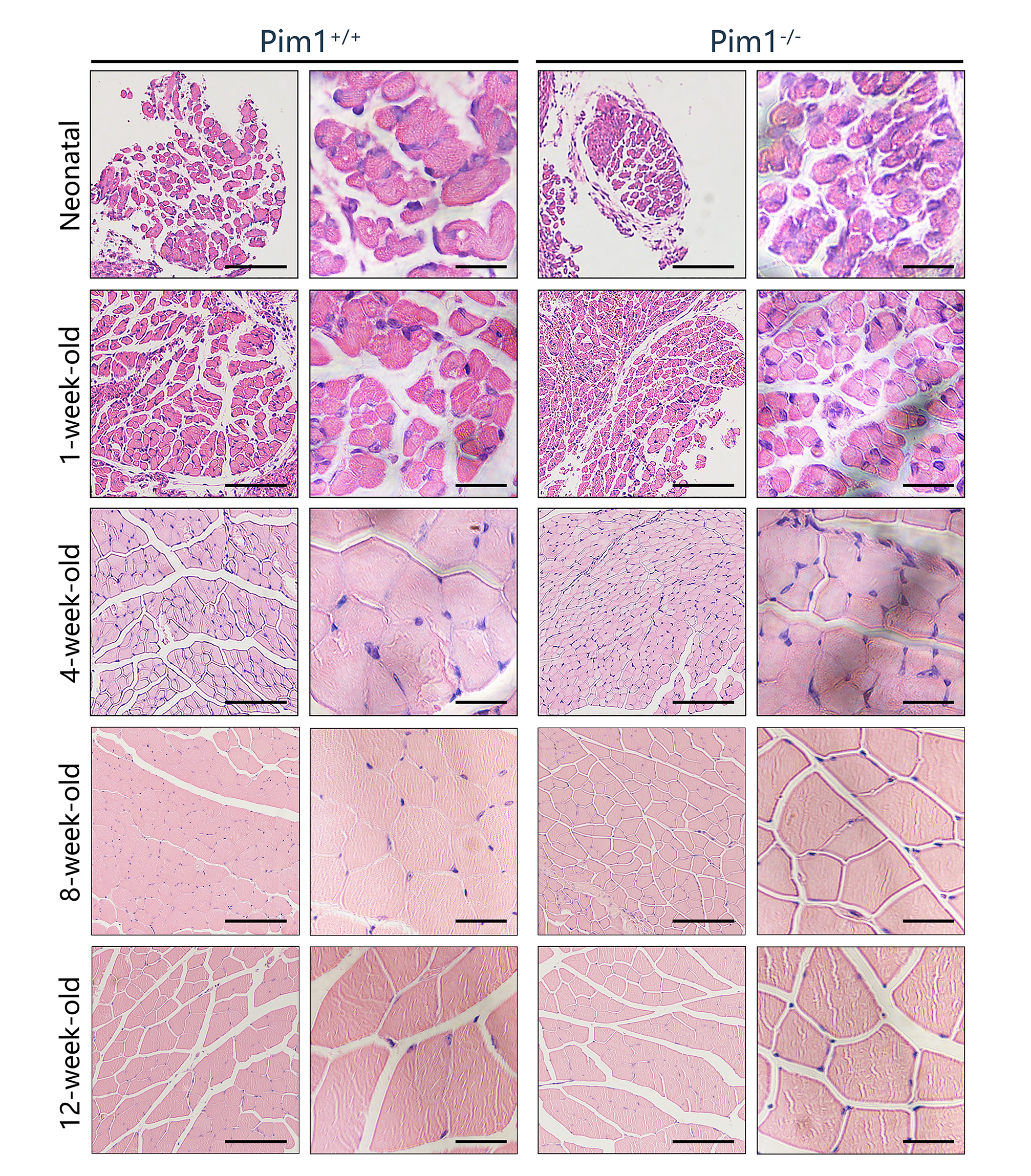

Supplement: Supplementary file 4 — Fig S3 [file 41419_2019_1993_MOESM4_ESM.tif]

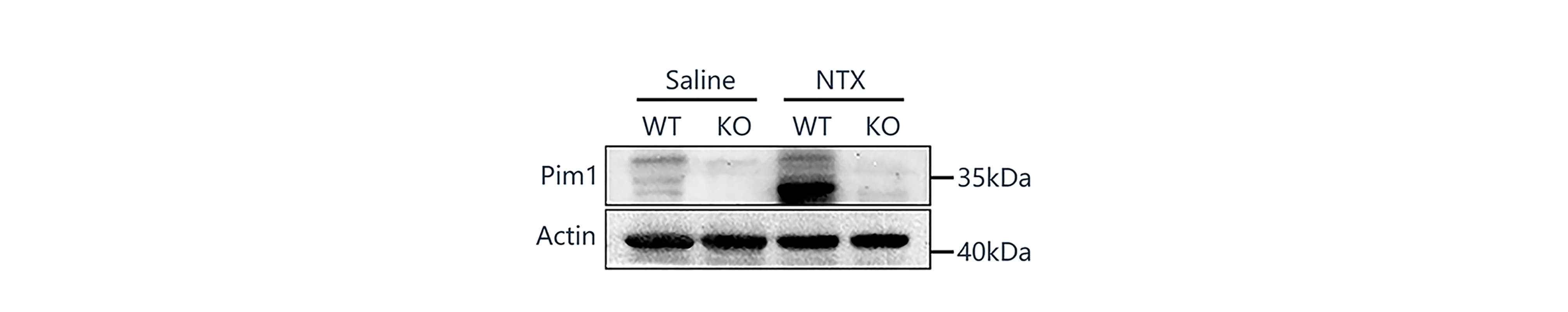

Supplement: Supplementary file 5 — Fig S4 [file 41419_2019_1993_MOESM5_ESM.tif]

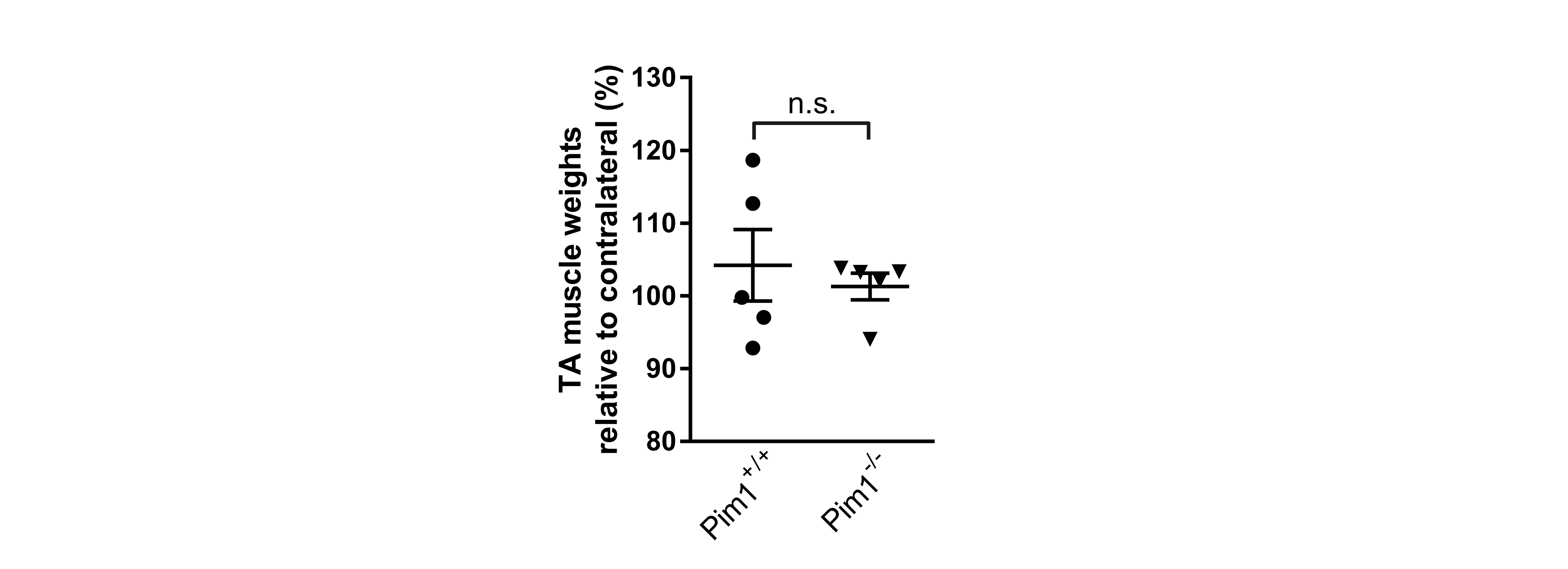

Supplement: Supplementary file 6 — Fig S5 [file 41419_2019_1993_MOESM6_ESM.tif]
