# Supplementary material for: Pim1 kinase positively regulates myoblast behaviors and skeletal muscle regeneration
Source: Cell Death Dis. 2019 Oct 10;10(10):773. doi: 10.1038/s41419-019-1993-3 (PMC6787030; doi:10.1038/s41419-019-1993-3)
Supplement: Supplementary file 7 — Table S1 [file 41419_2019_1993_MOESM7_ESM.docx]

**Table S1.** Mouse qPCR primers

Mouse qPCR primers

| Gene | Forward | Reverse |
| --- | --- | --- |
| *Pim1* | 5’-CATCAAGGGCCAAGTGTTCT-3’ | 5’-GATGGTTCCGGATTTCTTCA-3’ |
| *Myog* | 5’-ACCCTACAGACGCCCACAATC-3’ | 5’-GAAGGCAACAGACATATCCTCCAC-3’ |
| *Tnni2* | 5’-ACTTCGGAGGGTGCGTATGTC-3’ | 5’-CGTTCCTTCTCAGTGTCTTCCTTC-3’ |
| *Ckm* | 5’-CAGACCTCAGCAAGCACAACAATC-3’ | 5’-ATGACATCGTCCAGAGTGAAGCC-3’ |
| *Mylpf* | 5’-TCAGAACAGGGATGGCATTATCG-3’ | 5’-CAGGAAGACAGTGAAGTTGATGGG-3’ |
| *Acta1* | 5’-CGGCATCGTGTTGGATTCTGG-3’ | 5’-GGCGTGTGGCAGGGCATAG-3’ |
| *Mymk* | 5’-CTGGTTTGTCTGTGCTGTGCTTC-3’ | 5’-TGCTGGGTGTAGATGCTCTTGTC-3’ |
| *Mymx* | 5’-ACCAGCTTTCATGCCAGAAG-3’ | 5’-ATGTCTTGGGAGCTCAGTCG-3’ |
