# Supplementary material for: Pim1 kinase positively regulates myoblast behaviors and skeletal muscle regeneration
Source: Cell Death Dis. 2019 Oct 10;10(10):773. doi: 10.1038/s41419-019-1993-3 (PMC6787030; doi:10.1038/s41419-019-1993-3)
Supplement: Supplementary file 8 — Supplemental Fig Legends - clean [file 41419_2019_1993_MOESM8_ESM.doc]

**Supplemental Fig Legends**

**Fig. S1** Expression and quantification of the MyoD protein in C2C12 myotubes after TCS (25μM, 50μM) treatment for 72h (n = 8). GAPDH served as the internal reference. The data are shown as mean ± SEM. Independent-samples *t* test. ****P* < 0.001; n.s. = not significant

**Fig. S2** Confocal images showing the colocalization between Pim1 (green) and MyoD (red) in the nucleus (blue, DAPI-labelled) of primary myotube on 4d post-differentiation. Scale bar = 10μm.

**Fig. S3** H&E staining of the lower limb muscle transverse section of *Pim1*^+/+^ and *Pim1*^-/-^ mice from birth to 12-week-old. Scale bar = 100μm for the left column of each genotype; Scale bar = 20μm for the right column of each genotype.

**Fig. S4** Expression of the Pim1 protein in isolated TA muscles from NTX treated *Pim1*^+/+^ and *Pim1*^-/-^ mice. Actin served as the internal reference.

**Fig. S5** Percentage of the TA muscle weights relative to contralateral in *Pim1*^+/+^ and *Pim1*^-/-^ mice at 14 days after NTX injection (n = 5). The data are shown as mean ± SEM. Independent-samples *t* test. n.s. = not significant
